# Intraoperative Injection *vs* Sponge-applied Mitomycin C during Trabeculectomy: One-year Study

**DOI:** 10.5005/jp-journals-10028-1233

**Published:** 2017-10-27

**Authors:** Albert S Khouri, Grace Huang, Linda Y Huang

**Affiliations:** 1 Associate Professor, Department of Ophthalmology, Rutgers University, Newark New Jersey, USA; 2 Resident Physician, Department of Ophthalmology, Icahn School of Medicine Mount Sinai, New York, USA; 3 Fellow, Department of Ophthalmology, The Bascom Palmer Eye Institute, Miami, Florida, USA

**Keywords:** Antimetabolite, Glaucoma filtration surgery, Mitomycin C, Subconjunctival injection, Trabeculectomy.

## Abstract

**Aim:**

To determine the safety and efficacy of intraoperative injection of mitomycin C (MMC) against conventional sponge-applied MMC during trabeculectomy.

**Materials and methods:**

This study was a retrospective, comparative case series. Thirty eyes with primary open-angle glaucoma underwent consecutive trabeculectomies with MMC injection (injection group), and thirty eyes with sponge-applied MMC were as controls (sponge group). Data were collected preoperatively and postoperatively at 1 day, 1 week, 1 month, 3 months, 6 months, and 1 year after surgery. Demographic data, applanation intraocular pressure (IOP), best-corrected visual acuity (VA), number of glaucoma medications, postoperative interventions, postoperative complications, and number of visits within 3 months were recorded. In order to stratify data, proportion of eyes achieving >30% IOP reduction from baseline with or without glaucoma medications was calculated and defined as surgical success.

**Results:**

Mean IOP reduction at 1 year was significant in both the injection and sponge groups from baseline (46.8 and 37.8% respectively). The injection group had overall lower postoperative IOP and comparable complete treatment success, defined as achieving >30% IOP reduction without glaucoma medications (p = 0.941). The number of postoperative visits within 3 months and the proportion of eyes needing 5-fluorouracil (5-FU) intervention were significantly lower in the injection group (p = 0.03, p = 0.04 respectively).

**Conclusion:**

Injection of MMC was as safe and effective as sponge application with comparable estimated complete treatment success, less need for visits within 3 months, and 5-FU intervention.

**Clinical significance:**

Surgeons may consider intraopera-tive injection of MMC in appropriate patient cohorts given comparable safety and efficacy and several advantages over traditional sponge application. Further study in a prospective, larger, long-term manner is necessary to assess this modality.

**How to cite this article:** Khouri AS, Huang G, Huang LY. Intraoperative Injection *vs* Sponge-applied Mitomycin C during Trabeculectomy: One-year Study. J Curr Glaucoma Pract 2017;11(3):101-106.

## INTRODUCTION

Mitomycin C is an antineoplastic/antibiotic agent isolated from soil bacterium Streptomyces caespitosus. It acts as a deoxyribonucleic acid cross-linker, which inhibits fibroblast proliferation. It is used widely in medicine as a chemotherapeutic agent to treat a variety of cancers. Its use and application in ophthalmology is common practice because of its modulatory effects on wound healing.^1^ Current applications of MMC include glaucoma surgery, pterygium surgery, corneal refractive surgery, cicatricial eye disease, conjunctival neoplasia, and allergic eye disease.^2^

For more than two decades, MMC has been routinely used during trabeculectomy to reduce postoperative episcleral fibrosis and bleb failure due to scarring by the wound healing process.^3^ It was found to be effective in inhibiting fibroblastic activity, and its use has tremendously impacted the success rates of trabeculectomy.^4^ The use of MMC in trabeculectomy is indicated in patients who are young, African-American, or have had previous surgery, and has been shown to increase fibroblast density and compact connective tissue over time.^5^ Studies have shown that the use of MMC improves outcomes in glaucoma filtration surgery with good long-term IOP control.^6-8^ Traditionally, MMC is applied by being soaked onto a surgical sponge and placed onto the scleral surgical site prior to creation of the ostomy, before or after formation of a partial thickness scleral flap. The sponge is removed after a variable amount of time depending on the surgeon’s preference, ranging from 30 seconds to 5 minutes.^9^

Frequently, MMC has also been used as a subconjunctival injection before needle revision of failing filtering blebs. This method has been shown to be both safe and effective.^3,10-14^ Recently, there has been a trend toward subconjunctival injection of MMC during glaucoma filtration surgery. The purpose of this study was to determine the safety and efficacy of intraoperative injection of MMC against conventional sponge-applied MMC during trabeculectomy. A prior study looking only at a MMC injection group of trabeculectomies found the technique to be effective;^15^ however, our study is the first published comparative case series on this topic using a sponge-applied MMC group as a control.

## MATERIALS AND METHODS

### Study Design

This study was a retrospective, comparative case series designed from a consecutive series of trabeculectomies with MMC performed in a single center by one surgeon (A.S.K.) with the same standardized technique. Inclusion criteria were trabeculectomies with MMC for IOP control in eyes with primary open-angle glaucoma with follow-up of at least 3 months. The study group (injection group) included all trabeculectomies that met the above inclusion criteria and were performed consecutively between March 2013 and January 2014 (n = 30). The control group (sponge group) was selected from trabeculectomy procedures performed by the same surgeon between February 2010 and August 2013 that met the inclusion criteria and was matched for baseline IOP and VA (n = 30). Exclusions were patients undergoing any glaucoma procedure other than glaucoma filtration surgery with MMC, use of an antimetabolite, such as 5-FU, tube-shunt procedures, nonpenetrating glaucoma surgery, combination surgery (i.e., phacoemulsification + trabeculectomy), and any patients with a diagnosis other than primary open-angle glaucoma (i.e., uveitic, neovascular, traumatic glaucoma). This study was approved by the Institutional Review Board/ Ethics Committee of Rutgers New Jersey Medical School and adheres to the tenets of the Declaration of Helsinki.

### Trabeculectomy Outcomes

Data were collected preoperatively and postoperatively at 1 day, 1 week (±3 days), 1 month (±2 weeks), 3 months (±6 weeks), 6 months (±8 weeks), and 1 year (±16 weeks) after surgery. Additional visits were added as indicated. Demographic data and burden of postoperative care (number of visits within 3 months) were recorded. Baseline IOP and VA were calculated using the average of measurements from the two most recent visits prior to surgery. Goldmann applanation IOP, best-corrected VA, number of glaucoma medications, the need for postoperative interventions, and postoperative complications were recorded at each examination. Specifically, postoperative data on complications including bleb leak, hypotony (defined as IOP <6 mm Hg), shallow AC (defined as iris/cornea touch beyond the mid-iris centrally), infection, corneal edema/haze, and cataract formation were collected.

### Operative Procedures

**Fig. 1:**
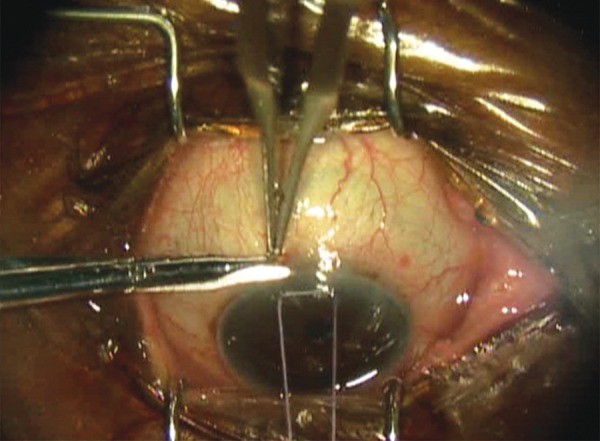
Snip peritomy performed with a limbal incision during trabeculectomy

**Fig. 2:**
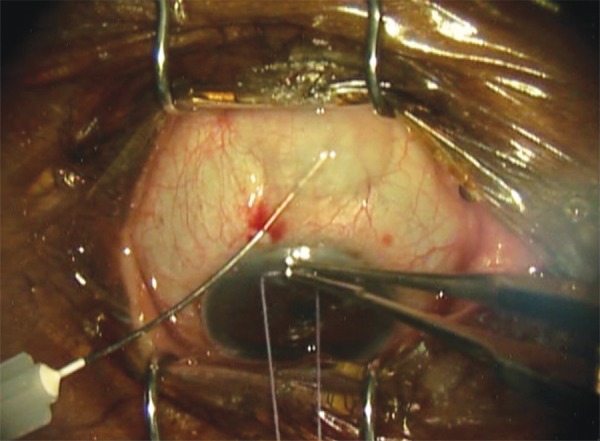
MMC preparation injected at the operative site during trabeculectomy

**Fig. 3:**
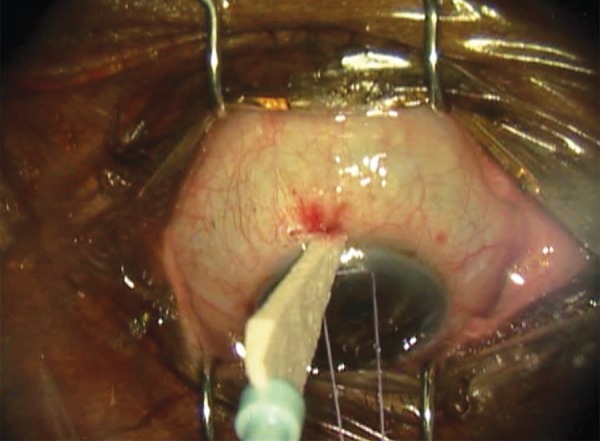
Compression of the conjunctival entry with a surgical sponge to avoid egress of MMC to the surface during trabeculectomy

All trabeculectomies were performed at a single institution by a single surgeon (A.S.K.). Dosage was adopted from dosing used in needle revision.^14^ To prepare the MMC injection, the surgeon used a 20-ug preparation starting with MMC 0.4 mg/mL, diluting 0.1 mL of MMC (40 μg) in 0.1 mL of lidocaine (1:1, total volume of 0.2 mL). Half of that solution (0.1 mL of MMC:lidocaine [20 μg]) was used for injection. Topical anesthesia was instilled. Snip peritomy was performed with a limbal incision (Fig. 1). A blunt 30-gauage cannula was introduced 7 to 8 mm from the limbus. The MMC preparation was injected posterior to the anticipated flap location subconjuncti-vally (Fig. 2). In order to avoid egress to the surface, the incision was kept small and the conjunctival entry was compressed with a surgical sponge to prevent any MMC from escaping (Fig. 3). The blunt cannula was withdrawn, and the solution was further spread over a larger surface area using a surgical sponge. The conjunctival peritomy was then completed. Wet-field bipolar cautery was performed for hemostasis with copious irrigation using balanced salt solution. The trabeculectomy was completed in the standard fashion by delineating a 3 × 3 mm scleral flap using a diamond knife preset at 300 μm. A 57 blade was then used to dissect the partial thickness scleral flap. A paracentesis was performed using a 1-mm side port blade in the temporal cornea. A sclerotomy was created with a Kelly punch. A peripheral iridectomy was created with a DeWecker scissors. The scleral flap was repositioned in place using two 10-0 nylon fixed sutures (nonreleasable) at the corners of the scleral flap. Balanced salt solution was injected in the anterior chamber, and flow through the trabeculectomy site was confirmed by the surgeon using surgical sponges. If the flow was too brisk, additional sutures were placed. Once flow was determined to be adequate, with the anterior chamber remaining well maintained, conjunctival closure proceeded using a running 9-0 nylon suture on a vascular needle. At the end of the case, the conjunctival incision was checked for lack of leakage.

The conventional sponge-applied technique was used in the control group. On two separate semicircular surgical sponges (7-mm corneal light shield cut in half), a MMC solution of 0.4 mg/mL was used and then inserted subconjunctivally at the surgical site. The sponges were applied for 2 minutes and removed, and then the area was copiously irrigated with balanced salt solution before the case proceeded in the usual fashion as described earlier.

### Statistical Analysis

Means and standard deviations were calculated for appropriate variables. For quantitative variables, a Student’s t-test was used to compare the means. Visual acuity was converted to logarithm of the minimum angle of resolution units before statistical analysis. Qualitative variables were analyzed using the chi-square test and Fisher’s exact test. Statistical analysis was performed using Microsoft Excel 2011 (Redmond, Washington); p-values of 0.05 or less were considered to be statistically significant. In order to stratify surgical success, we calculated proportion of eyes achieving >30% IOP reduction from baseline with and without the use of glaucoma medications. Complete success was defined as >30% IOP reduction from baseline without the use of glaucoma medications. Qualified success was defined as >30% IOP reduction from baseline with or without the use of glaucoma medications. Kaplan-Meier analysis was performed using XLSTAT (Copyright 2015, Addinsoft) with percent survival defined as complete treatment success. End points in our study were “Loss of light perception” which developed in any patient as a direct complication posttrabeculectomy or “Failure,” defined as any patients that needed additional glaucoma surgery, had sustained elevation in IOP above 22 mm Hg for more than 4 weeks on maximally tolerated medical therapy, and/or had a devastating complication, such as endophthalmitis.

## RESULTS

**Graph 1:**
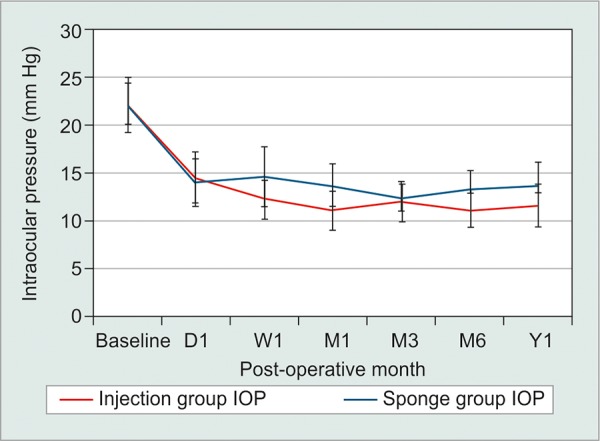
Intraoperative injection *vs* sponge-applied MMC during trabeculectomy: Postoperative IOP over time

**Graph 2:**
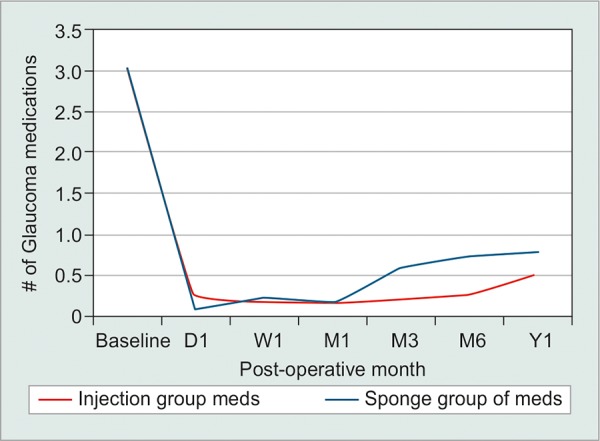
Intraoperative injection *vs* sponge-applied MMC during trabeculectomy: Postoperative number of medications over time

**Graph 3:**
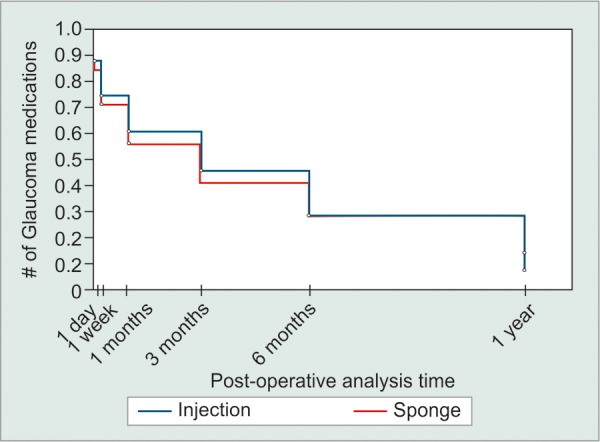
Intraoperative injection *vs* sponge-applied MMC during trabeculectomy: Kaplan-Meier survival plot. Success was defined as an IOP reduction of at least 30% from preoperative values, without glaucoma medications (p = 0.941)

In total, 60 eyes were included: 30 intraoperative MMC injection and 30 sponge-applied MMC. There were eight patients in the injection group and three patients in the sponge group with previous cataract surgery. None of the patients had previous incisional glaucoma surgery. In total, three patients had bilateral trabeculectomies. Two patients with bilateral surgery had one eye assigned to the injection group and one eye to the sponge group. A single patient in the injection group had surgery in both eyes. There were no differences in baseline IOP, VA, age, or number of glaucoma medications between groups (p > 0.05). Mean IOP reductions from baseline were significant in both groups at each time point (p < 0.05). Mean IOP reduction from baseline was 46.8% in the injection group and 37.8% in the sponge group at 1 year. There were no significant differences at any time point in postoperative IOP, VA, number of glaucoma medications, or complications when comparing outcomes between groups (p > 0.05; Table 1). Although the injection group had overall lower mean IOP and lower mean number of glaucoma medications, this did not reach significance (p > 0.05; Graphs 1 and 2). Overall complete treatment success was 63.6% in the MMC injection and 44% in the MMC sponge group at postoperative year 1. Kaplan-Meier analysis revealed comparable estimated complete treatment success between the injection and sponge group (p = 0.941; Graph 3).

**Table 1:** **Table 1:** Intraoperative injection *vs* sponge-applied MMC during trabeculectomy: mean IOP and number of medications

		*Baseline* *(I = 30, S = 30)*		*D1* *(I = 29, S = 29)*		*W1* *(I = 29, S = 29)*		*M1* *(I = 29, S = 27)*		*M3* *(I = 30, S = 25)*		*M6* *(I = 25, S = 24)*		*Y1* *(I = 22, S = 25)*	
IOP		Injection 21.9 ± 7.73		14.5 ± 7.18		12.4 ± 6.14		11.1 ± 5.53		12 ± 6.02		11.1 ± 4.66		11.7 ± 5.43	
		Sponge 22.1 ± 8.14		13.9 ± 6.93		14.6 ± 8.6		13.7 ± 5.87		12.4 ± 3.62		13.3 ± 4.71		13.7 ± 6.22	
# of meds		Injection 3.03 ± 1.25		0.23 ± 0.73		0.17 ± 0.40		0.17 ± 0.60		0.24 ± 0.79		0.28 ± 0.54		0.53 ± 0.77	
		Sponge 3.03 ± 1.27		0.10 ± 0.31		0.23 ± 0.63		0.17 ± 0.39		0.60 ± 1.15		0.74 ± 1.14		0.80 ± 1.29	

Meds: Medications; I: Injection; S: Sponge; D1: Postoperative day 1, W1: Postoperative week 1, M1: Postoperative month 1, M3: Postoperative month 3, M6: Postoperative month 6, Y1: Postoperative year 1

There was no difference between groups in postoperative complications including bleb leak, hypotony, shallow AC, infection, corneal edema/haze, and cataract formation (p > 0.05; Table 2). Specifically, there was no difference in postoperative bleb leak or hypotony between groups (p > 0.05; Table 2). No patients in either group had persistent corneal edema/haze postoperatively.

There was no significant difference between the proportions of eyes needing postoperative laser suture lysis (LSL) intervention. The proportion of eyes needing 5-FU postoperative intervention in the injection group was significantly lower than that of the sponge group (p = 0.04; Table 2). In the injection group, there were a total of five patients that received 5-FU. The greatest number of 5-FU administrations was between postoperative week 1 and month 1 (Table 3). In the sponge group, there were a total of 13 patients that had received 5-FU, with the greatest number of administration also occurring during postoperative week 1 and month 1 (Table 3). The decision to perform postoperative interventions was made at the discretion of the surgeon taking into consideration when the IOP was not on target, visual fields, the characteristic of the nerve, or when bleb morphology was unfavorable (i.e., encapsulated).

The burden of postoperative care (mean number of visits within 3 months) was also significantly less in the injection group compared with the sponge group (5.87 and 7.32 respectively, p = 0.03). One patient in the injection group needed additional surgery or revisions, whereas three patients in the sponge group needed a second glaucoma surgery. No patients in either group developed loss of light perception vision or were “Failures,” defined as having sustained elevation in IOP above 22 mm Hg with medications for more than 4 weeks or had a devastating complication, such as endophthalmitis.

**Table 2:** **Table 2:** Intraoperative injection *vs* sponge-applied MMC during trabeculectomy: postoperative interventions and complications

		*Proportion of eyes (n = 30)*					
		*Injection*		*Sponge*		*p-value*	
5-FU		0.17		0.43		0.047*	
LSL		0.30		0.50		0.114	
Bleb leak or hypotony		0.26		0.20		0.541	
Postoperative complications		0.27		0.30		0.774	
Additional surgery/re-op		0.03		0.10		0.300	

*Statistically significant

**Table 3:** **Table 3:** Number of eyes receiving 5-FU and LSL intervention after trabeculectomy

				*D1 (I = 29,* *S = 29)*		*W1 (I = 29,* *S = 29)*		*M1 (I = 29,* *S = 27)*		*M3 (I = 30, S = 25)*		*M6 (I = 25,* *S = 24)*		*Y1 (I = 22,* *S = 25)*	
5-FU		Injection		0		*3*		4*		*2*		0		1	
		Sponge		0		8		15*		2		1		1	
LSL		Injection		0		7		2		0		1		0	
		Sponge		1		13		7		0		0		0	

*Statistically significant, p = 0.003; I: Injection; S: Sponge; D1: Postoperative day 1; W1: Postoperative week 1; M1: Postoperative month 1; M3: Postoperative month 3, M6: Postoperative month 6; Y1 = postoperative year 1

## DISCUSSION

Our study shows that the efficacy of injection of MMC is comparable to sponge application, with less need for visits within 3 months, and 5-FU intervention. Overall complete treatment success in the MMC injection group at 1 year was 64%, which is consistent with a prior noncompara-tive study reporting 1-year outcomes of MMC injection in trabeculectomy using the same measure of success.^15^ Intraoperative injection of MMC in trabeculectomy has several advantages over conventional sponge application. One benefit it provides is a large surface area of exposure. A large MMC treatment area produces more diffuse and elevated blebs.^16^ Large-area MMC application also seems to increase long-term success without increasing the complication rates in trabeculectomies.^17,18^ An animal study showed that the size of the area of subconjunctival MMC treatment significantly affects surgical outcome with small areas of treatment producing thin-walled and localized blebs with significant short-term scarring.^16^ Direct and diffuse application of MMC by injection may promote less scarring and vascularization of the bleb.^19^ In order to achieve the same surface area of exposure with sponges, i.e., achieved with injection, the surgeon must use multiple sponges, all of which must be carefully collected thereafter. The injection method therefore, eliminates the risk of retained sponges.

Another advantage of using injection *vs* sponge application of MMC is the predictable dose of delivery. In sponge application, the surface area of cut pieces of surgical sponges is very variable. A study found that the quantities of MMC contained in sponges prepared for glaucoma surgery differed for a given surgeon and between surgeons. The estimated actual dose delivered in a sponge soaked with MMC 0.2 mg/mL varied between 1.9 and 17.3 μg.^20^ With this unpredictable sponge dosing, surgeons run the risk of overdosing MMC. Irrigation is often used after delivery of MMC; however, it appears to only have an effect at reducing MMC concentrations in the superficial scleral layers, with no effect on MMC concentrations in the deep scleral and subscleral layers.^21^ Regardless of the device used, MMC seems to penetrate intraocularly with the highest variability of remaining MMC concentration found in the surgical sponge delivery method.^22^

A possible dose-response relationship seems to exist between the concentration of and duration of exposure to MMC.^23^ The main complications and side effects of MMC-enhanced filtration surgery are comprised of late bleb leaks, bleb infections, endophthalmitis, chronic hypotony, hypotony maculopathy, and corneal epithelial toxicity.^3^ Hypotony and its sequelae may be related to intraocular toxicity of MMC.^22^ Occasionally, sponge application can also create a whitish MMC “burn” often due to overdosing of MMC. The avascular, thin bleb produced is at increased risk of early and late bleb leaks as well as of infection. These localized filtering blebs tend to be functionally limited by encapsulation and sequestration within what is classically described as a “ring of steel” (i.e., surrounding Tenon fibrosis). Our study shows that the injection of MMC is safe, with fewer need for postoperative 5-FU intervention and burden of care (number of visits within 3 months; p = 0.04, p = 0.03 respectively). Patients were asked to come in for additional visits at the clinician’s discretion based on the eye examination and the need for additional interventions (i.e., postoperative care, 5-FU, LSL). The significance of fewer visits within 3 months in the injection group may be due to more favorable bleb morphology, which necessitated fewer interventions and therefore, fewer clinic visits.

A prior noncomparative study of MMC injection found that the most frequent early postoperative complications were hypotony, hyphema, and serous choroidal detachments.^15^ In our study, there was no difference in postoperative complications between injection and sponge application. This is consistent with a single report on intra-Tenon injection of MMC during trabeculectomy that showed the injection group had a similar result and also had lower mean IOP and need for fewer glaucoma medications.^19^

This is the first comparative case series to be published on this topic. Limitations of this study include its retrospective design, relatively small sample size, and follow-up limited to 1 year. Further study in a prospective, long-term, larger cohort is necessary to further assess the efficacy and safety of this modality. Additional data may be collected including standardized bleb morphology grading, endothelial cell counts, and corneal thickness.

## CONCLUSION

In conclusion, injection of MMC may be as safe and as effective as conventional sponge application of MMC with comparable estimated complete treatment success, less need for 5-FU intervention, and burden of care.

## CLINICAL SIGNIFICANCE

Surgeons may consider intraoperative injection of MMC in appropriate patient cohorts given comparable safety and efficacy and several advantages over traditional sponge application.
